# Leucine alters hepatic glucose/lipid homeostasis via the myostatin-AMP-activated protein kinase pathway - potential implications for nonalcoholic fatty liver disease

**DOI:** 10.1186/1868-7083-6-27

**Published:** 2014-11-18

**Authors:** Aida Zarfeshani, Sherry Ngo, Allan M Sheppard

**Affiliations:** Developmental Epigenetics Group, Liggins Institute, The University of Auckland, 85 Park Road, Grafton Auckland, 1023 New Zealand

**Keywords:** leucine, myostatin, AMP-activated protein kinase, miRNAs, fatty liver

## Abstract

**Background:**

Elevated plasma levels of the branched-chain amino acid (BCAA) leucine are associated with obesity and insulin resistance (IR), and thus the propensity for type 2 diabetes mellitus development. However, other clinical studies suggest the contradictory view that leucine may in fact offer a degree of protection against metabolic syndrome. Aiming to resolve this apparent paradox, we assessed the effect of leucine supplementation on the metabolism of human hepatic HepG2 cells.

**Results:**

We demonstrate that pathophysiological leucine appears to be antagonistic to insulin, promotes glucose uptake (and not glycogen synthesis), but results in hepatic cell triglyceride (TG) accumulation. Further, we provide evidence that myostatin (MSTN) regulation of AMP-activated protein kinase (AMPK) is a key pathway in the metabolic effects elicited by excess leucine. Finally, we report associated changes in miRNA expression (some species previously linked to metabolic disease etiology), suggesting that epigenetic processes may contribute to these effects.

**Conclusions:**

Collectively, our observations suggest leucine may be both ‘friend’ and ‘foe’ in the context of metabolic syndrome, promoting glucose sequestration and driving lipid accumulation in liver cells. These observations provide insight into the clinical consequences of excess plasma leucine, particularly for hyperglycemia, IR and nonalcoholic fatty liver disease (NAFLD).

## Background

The relative nutrient abundance that is associated with modern Western dietary patterns causes a rapid increase in postprandial plasma glucose and insulin levels and is associated with a propensity toward the development of metabolic syndrome characterized by visceral obesity, insulin resistance (IR) and type 2 diabetes mellitus (T2DM). The branched-chain amino acids (BCAAs) account for 15% to 25% of the total protein intake in the modern diet [1], and increased plasma levels are clinically associated with an obese phenotype [2] and progression to T2DM [3, 4]. Indeed, the levels of BCAAs are more strongly associated with IR than are many of the common circulating lipid species [2] and may even be predictive indicators of future T2DM risk [4]. However, whether elevated BCAAs directly promote progression of metabolic syndrome remains unclear, some studies even suggesting that the BCAA leucine offers a level of protection against IR, either by increasing muscle glucose utilization [5] or by energy expenditure in thermogenic tissues [6].

To address this somewhat contradictory clinical picture, we explored the phenotypic and molecular changes induced in hepatic cells following leucine supplementation. As previously reported for an *in vivo* study [7], we also report enhanced glucose uptake *in vitro*, a presumed benefit for limiting the onset of IR. However, we also find increased *de novo* hepatic lipogenesis and triglyceride (TG) deposition. Human and animal studies link high glycemic diets with increases in hepatic fat storage, steatosis and nonalcoholic fatty liver disease (NAFLD) [8] and the Western lifestyle of nutrient abundance and physical activity [9]. Thus, leucine is perhaps both ‘friend and foe’ in the context of metabolic syndrome. We also report central roles for myostatin (*MSTN)*-dependent AMP-activated protein kinase (AMPK) signaling and miRNA-dependent epigenetic processes in these metabolic effects.

## Results

### Leucine changes hepatic glucose and triglyceride homeostasis

**Figure 1:**
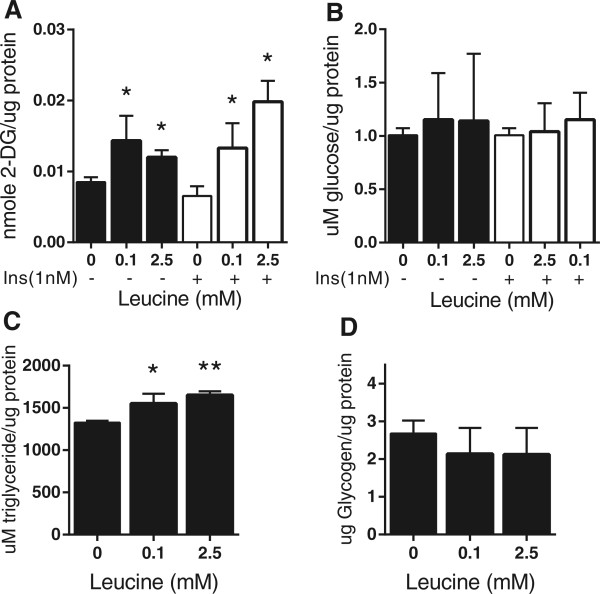
**Effect of leucine supplementation on glucose and lipid metabolism in HepG2 cells. (A)** 2-deoxy-D-[1,2-^3^H] glucose (2-DOG) consumption with or without the addition of insulin (1nM). Total intracellular glucose uptake was normalized to total protein content. **(B)** Levels of glucose secreted into the media with or without addition of insulin (1nM). **(C)** Total intracellular amount of triglyceride (TG), normalized to total protein content. **(D)** Glycogen content of homogenized samples treated with glucoamylase to hydrolyze glycogen into glucose. Glycogen amount was normalized to total protein content. Values are presented as mean ± SEM. Statistical significance relative to untreated control, *P <0.05, **P <0.01 (n =3).

To examine the effect of increased leucine on hepatic glucose utilization, we first assessed uptake by HepG2 cells. Compared to untreated controls, basal 2-deoxy-D-[1,2-^3^H] glucose (2-DOG) uptake was significantly increased (25% and 33%) with 0.1 mM and 2.5 mM leucine, respectively (*P* ≤0.05; Figure 1A). Interestingly, insulin-stimulated 2-DOG uptake was further enhanced (50% and 71%) in the presence of leucine (*P* ≤0.05), indicating that leucine may augment glucose utilization independent of insulin. As we found no evidence that leucine stimulated glucose secretion (Figure 1B), we suggest that its primary effect on hepatocyte glucose homeostasis is to enhance sequestration.

We questioned whether this increase in glucose uptake resulted in enhanced conversion to lipids by measuring total intracellular TG, which increased significantly (16% and 21%) at 0.1 mM and 2.5 mM of leucine respectively compared to the untreated control (*P* ≤0.05; Figure 1C). Moreover, cellular glycogen was unchanged after leucine supplementation (Figure 1D).

### Leucine changes hepatic expression of glucose/lipid sensing genes

**Figure 2:**
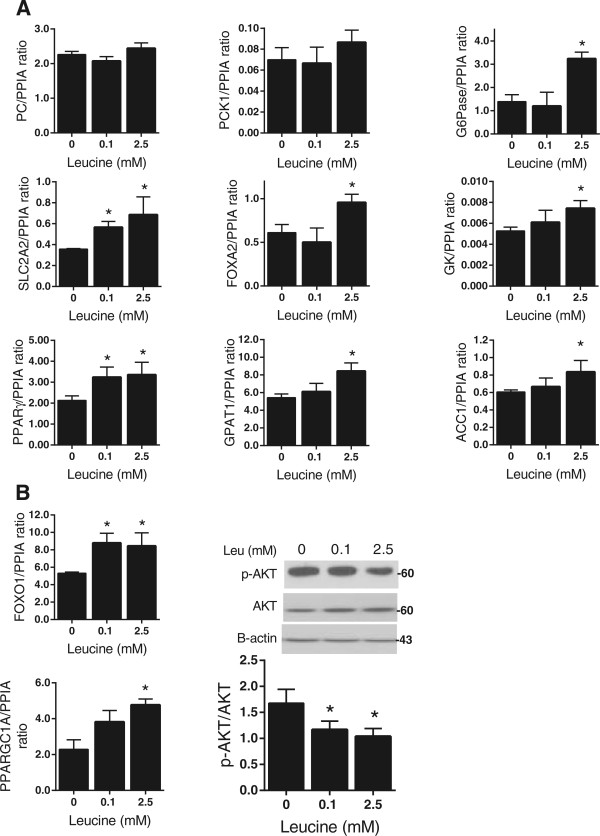
**Expression of the genes involved in glucose and lipid sensing after leucine supplementation of HepG2 cells. (A)** Real-time PCR (qPCR) and immunoblotting analysis of expression levels of genes involved in gluconeogenesis, glucose uptake and lipogenesis. **(B)** qPCR and immunoblotting analysis of specific upstream genes involved in glucose/lipid sensing. Western blotting was performed using cell lysates supplemented with leucine for 48 h. Values are presented as mean ± SEM. Statistical significance relative to untreated control, *P <0.05 (n =3).

Excessive hepatic glucose uptake is likely to contribute to the development of obesity-related dyslipidemia. To provide molecular evidence for the role of leucine in perturbing hepatic metabolism, mRNA expression levels of several key genes involved in lipid and glucose sensing were measured. The expression of pyruvate carboxylase *(PC)*, a ligase that catalyzes the carboxylation of pyruvate to oxaloacetate [10]; phosphoenolpyruvate carboxykinase (*PCK1; PEPCK),* which decarboxylates and phosphorylates oxaloacetate into phosphoenol pyruvate [10]; and glucose 6-phosphatase (*G6Pase*)*,* which catalyzes the final steps of gluconeogenesis, resulting in production of glucose [11], were measured. Although *PC* and *PCK1* expression remained unchanged, G6-Pase increased by 61% (*P* ≤0.05) at 2.5 mM leucine compared to the control (Figure 2A). In addition, the mRNA level of solute carrier family member2 (*SLC2A2)* was increased (*P* ≤0.05) by 34% at 0.1 mM and 46% at 2.5 mM of leucine. Moreover, peroxisome proliferative activated receptor-γ co-activator 1 (*PPARγ)* expression, a stimulator of endogenous *SLC2A2* mRNA transcription and key regulator of the genes associated with steatosis liver [12], was enhanced by about 40% after the leucine treatment (*P* ≤0.05; Figure 2A). Furthermore, expression of forkhead box protein A2 (*FOXA2),* which synergistically increases the promoter activity of the *SLC2A2* gene [13], was also increased by 33% (*P* ≤0.05; Figure 2A). We also found a significant increase in glucokinase *(GK)*, 33.3% at 2.5 mM (*P* ≤0.05; Figure 2A).

Glycerol-3-phosphate acyltransferase *(GPAT1)* catalyzes glycerol synthesis and thus TG biosynthesis [14]. *GPAT1* expression was increased by 33% at 2.5 mM leucine (*P* ≤0.05; Figure 2A). Acetyl-CoA carboxylase *(ACC1)* catalyses long-chain fatty acid biosynthesis [15] and was increased by 26% at 2.5 mM leucine (*P* ≤0.05; Figure 2A). Collectively, our data indicate that leucine supplementation promotes hepatic lipid synthesis; however, they do not demonstrate that overexpression of *SLC2A2* and *GPAT1* alone mediate the effect of leucine on glucose uptake and triglyceride biosynthesis.

*PPARGC1A* and forkhead transcription factor *(FOXO1)* play important roles in glucose metabolism [16], and nuclear accumulation of the latter also stimulates TG synthesis [17]. Expression of both increased by 54% (*P* ≤0.05) and 37%, respectively (Figure 2B). Activated protein kinase B (AKT) phosphorylates *FOXO1* [18] to prevent nuclear translocation, yet we found that (Ser^473^)-AKT phosphorylation was reduced by about 30% at both 0.1 mM and 2.5 mM leucine, suggesting that *FOXO1* was not only upregulated but functionally activated by leucine.

**Figure 3:**
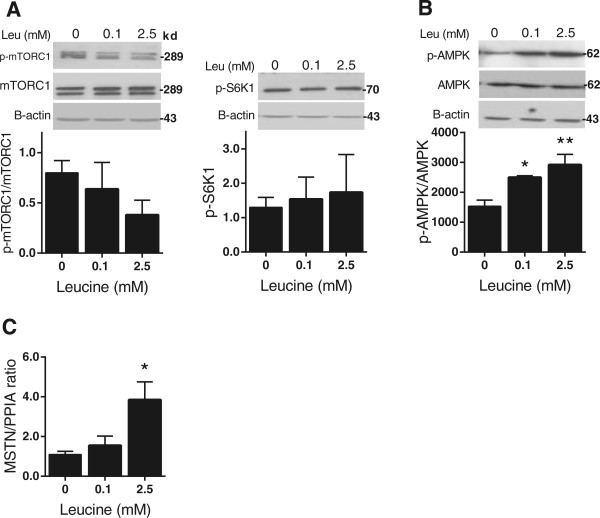
**AMP-activated protein kinase (AMPK) but not mammalian target of rapamycine complex 1/ Ribosomal protein S6 kinase beta-1 (mTOR/ S6K1) activity was regulated by leucine in HepG2 cells. (A)** Western blots and densitometry analysis of leucine-supplemented HepG2 cells stained for p-mTOR and p-S6K1 and **(B)** p-AMPK. **(C)** Effect of leucine supplementation on myostatin (MSTN) mRNA level. Values are the presented as mean ± SEM. Statistical significance relative to untreated control, *P <0.05, (n =3).

mTORC1 (mammalian target of rapamycine complex 1), a nutrient and hormonal sensor [19], regulates gene translation through phosphorylation and activation of ribosomal protein S6 kinase beta-1 (S6K1) [20]. We found either mTORC1 activity or S6K1 phosphorylation remained unchanged (Figure 3A). In addition to mTOR, AKT activity not only stimulates (Ser^2448^)-mTORC1 phosphorylation but also negatively regulates p(Thr^172^)- AMPK-α [21]. Leucine enhanced phosphorylation of (Thr^172^)- AMPK-α by 40% (*P* ≤0.05) and 50% (*P* ≤0.01) at 0.1 mM and 2.5 mM, respectively (Figure 3B).

Clinical obesity is associated with increased *MSTN* expression [22], and *MSTN* mRNA levels are increased in both adipose and skeletal muscle of obese mice [23]. We detected a fourfold increase in *MSTN* expression at 2.5 mM leucine (*P* ≤0.05) (Figure 3C).

### Myostatin is involved in the regulation of leucine modified genes

**Figure 4:**
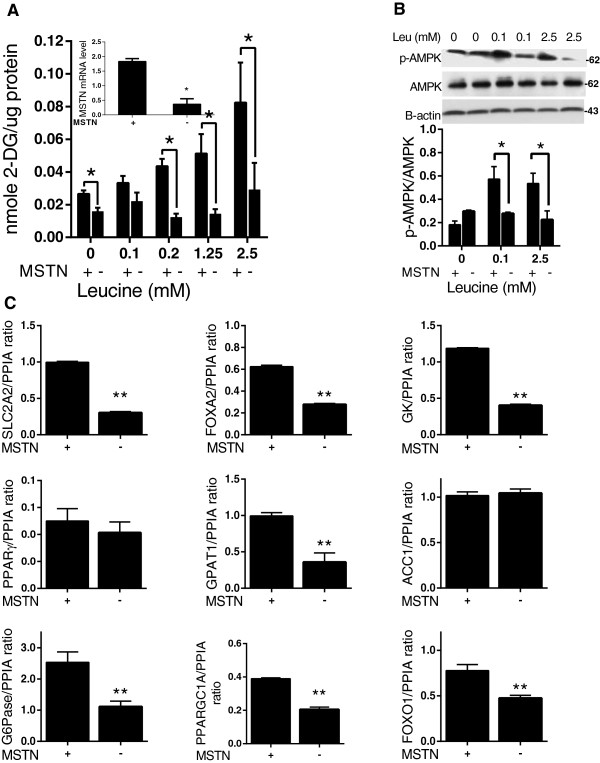
**Effects of myostatin (MSTN) on** AMP-activated protein kinase **(AMPK) activity and glucose /lipid sensing in leucine- treated HepG2 cells. (A)** Glucose uptake is repressed in MSTN-suppressed cells. MSTN knock-down efficiency using siRNA **(B)** The leucine- induced AMPK signaling pathway was suppressed following MSTN suppression. **(C)** mRNA levels of leucine-sensitive genes in the presence and absence of MSTN. Values are presented as the mean ± SEM. Statistical significance relative to untreated control, *P <0.05, **P <0.01 (n =3).

To determine the effect of MSTN on the cellular glucose uptake, we measured 2-DOG uptake in *MSTN* inhibited cells followed by leucine supplementation (Figure 4A). In the basal state, siRNA-mediated knockdown of *MSTN* led to a 40% (*P* ≤0.05) decrease in glucose uptake whereas in the presence of leucine, *MSTN* suppression led to 40 to 60% (*P* ≤0.05) reduction in glucose uptake across the various leucine doses, suggesting an *MSTN*-dependent effect of leucine on promoting glucose uptake. Next, we hypothesized that *MSTN* promoted leucine-mediated glucose uptake via AMPK activation and indeed found that *MSTN* knockdown decreased p(Thr^172^)-AMPK approximately 50% in the presence of both 0.1 mM and 2.5 mM leucine (*P* ≤0.05), while p(Thr^172^)-AMPK levels remained unchanged (with and without *MSTN*-knockdown) in the absence of leucine (Figure 4B). These results suggest that leucine-induced AMPK phosphorylation is mediated by *MSTN* signaling.

To identify which of the leucine-responsive genes were regulated by *MSTN*, we measured candidate mRNA expression following *MSTN* inhibition. As shown in Figure 4C, expression of most of them was markedly reduced (37% to 75%; *P* ≤0.01). *ACC1* and *PPARγ* were notable exceptions.

### miRNA array validation using quantitative real-time PCR

**Figure 5:**
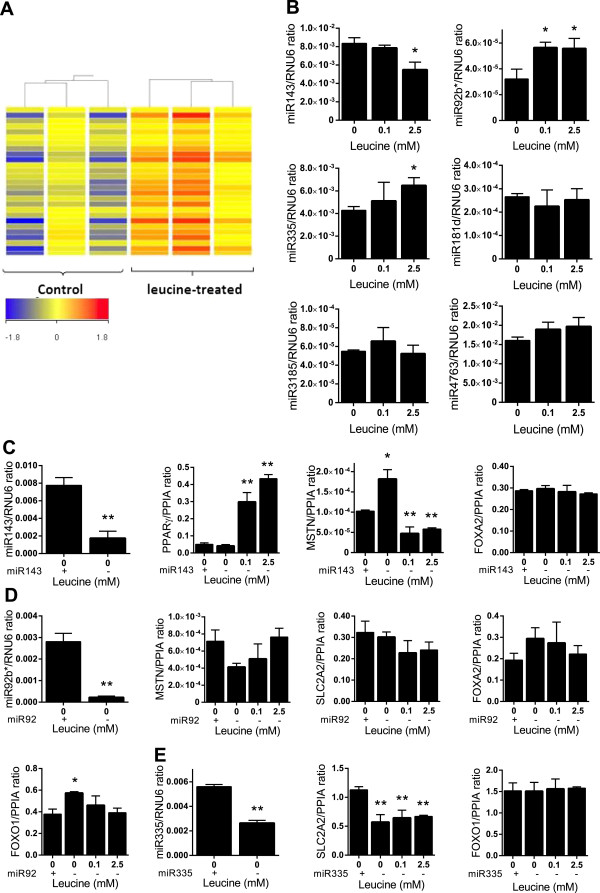
**Discovery and validation of miRNA expression after leucine supplementation. (A)** Cluster analysis of miRNAs available on Affymetrix miRNA chips. The red color shows relatively abundant expression of the same miRNA when compared to controls, whereas the blue color indicates a low expression relative to other samples. Cluster analysis was performed with Gene Spring GX. **(B)** Validation of specific miRNA species exhibiting significant difference from the control group, using real-time PCR (qPCR). Finally, differential expression of specific target genes measured after inhibition of miR-143 **(C)**, miR-92b*(D) and miR-335 **(E)** using selective siRNAs. Values are presented as the mean ± SEM. Statistical significance relative to untreated control, *P <0.05, **P <0.01 (n =3).

Hierarchical clustering using Pearson correlation identified 35 and 5 human miRNAs to be significantly up- or downregulated, respectively by 2.5 mM leucine compared to untreated controls (Figure 5A). We validated the expression of leucine-dependent microRNAs in HepG2 cells, including miR-143, miR-92b*, miR-335, miR-181d, miR-3185 and miR-4763 by real-time PCR (qPCR) (Table 1). As expected, the expression of miRNA-143 was reduced 1.5-fold (*P* ≤0.05), while that of miRNA-92b* and miR-335 was upregulated 1.8- and 1.5-fold (*P* ≤0.05) at 2.5 mM of leucine, respectively (Figure 5B). However, there was no significant difference in the expression of miR-181d, miR-3185 and miR-4763.

**Table 1:** **Real-time PCR (qPCR) validation of differentially expressed microRNAs in leucine-treated HepG2 cells compared with control**

***Probe set ID***	***Fold- Change***
hsa-miR-143	-1.52
hsa-miR-92b*	1.83
hsa-miR-335	1.51
hsa-miR-181d	-1.05
hsa-miR-3185	-1.04
hsa-miR-4763	1.22

To confirm whether up/downregulated miRNAs can modulate leucine’s effects on glucose and lipid metabolism, we measured the expression of leucine-dependent genes of interest in suppressed (or not) miRNAs. Following miR-143 suppression, leucine supplementation induced a significant increase in *PPARγ* expression by 6- and 8.6-fold at 0.1 mM and 2.5 mM leucine, respectively, compared to cells without miR-143 suppression (Figure 5C). This is in contrast to our earlier observation that leucine induced a modest 1.6-fold increase in HepG2 cells (without miR-143 suppression; Figure 2A), suggesting an inhibitory effect of miR-143 on *PPARγ* via leucine.

Also, we found increased *MSTN* level (1.8-fold; *P* ≤0.05) in the absence of leucine (Figure 5C). On the contrary, miRNA-143 suppression led to a reduced *MSTN* expression by 50% (*P* ≤0.01) in the presence of leucine, suggesting that *MSTN* is also regulated in a miR-143 dependent manner. Notably, leucine supplementation did not promote *FOXA2* expression (Figure 5C). Suppression of miR-92b* tended to reduce gene expression of *MSTN* in the absence of leucine but increased the *MSTN* level with the 2.5 mM leucine treatment, despite being statistically not significant (Figure 5D). Although not statistically significant, there was also a decrease in both *SLC2A2* and *FOXA2* expression, but only in the presence of leucine (Figure 5D). Also, *FOXO1* expression was increased by 1.5-fold (*P* ≤0.05) in the absence of leucine (Figure 5D). Finally, following miR-335 suppression, *SLC2A2* expression was reduced by 50% (*P* ≤0.01) in the presence and absence of leucine, but *FOXO1* expression was unchanged (Figure 5E). Collectively, these data indicate miRNAs influence key metabolic genes, either directly or in response to leucine.

## Discussion

**Figure 6:**
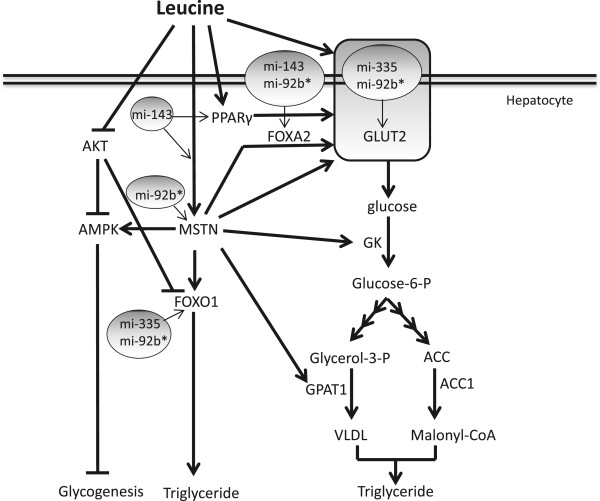
**Schematic summary of findings.** Leucine supplementation promotes hepatic cell glucose uptake by upregulating solute carrier family member2 (*SLC2A2*) expression via myostatin (*MSTN). MSTN* activity leads to the activation of AMP- activated protein kinase (AMPK) and inhibition of glycogen synthesis. Furthermore, activation of *MSTN* leads to overexpression of genes involved in glucose uptake, which is further responsible for triglyceride synthesis. Moreover, leucine supplementation alters the expression of several small RNA species including miR-143, miR-335 and miR-92b*, which target main gene regulators of these effects.

The intake of dietary protein (BCAAs) influences glucose metabolism and insulin sensitivity. Both IR and T2DM associated with metabolic syndrome often correlate with considerable changes in amino acid metabolism [24]. We show that elevated leucine increases glucose uptake by HepG2 cells and activates the glucokinase gene, the apical sensor of intracellular glucose levels (Figure 6). Further, we suggest this enhanced glucose uptake may be a consequence of increased *SLC2A2* gene transcription, resulting from increased expression of *FOXA2* a critical downstream effector of metabolic processes and transcription factor known to promote *SLC2A2* transcription [13]. Meanwhile, we do not see evidence for glucose secretion or for any change in the *PEPCK* and *PC* gene transcription, implying that leucine does not promote gluconeogenesis. However, we did note increased *G6Pase* transcription. Although often linked to gluconeogenesis, the key cellular role of *G6Pase* in buffering G6P concentrations is also dependent on the processes of glycolysis and glycogenolysis [11]. Further, we did not observe any increase in glycogen content, rather a tendency towards decreasing glycogen.

The primary regulation of systemic glucose levels by insulin is triggered when hepatic receptors recruit the insulin receptor substrate (IRS), leading to activation (phosphorylation) of PI3K/AKT and in turn phosphorylation of *FOXO1*, which blocks its nuclear translocation and inhibits activation of gluconeogenic genes (notably *PEPCK* and *PC*) to reduce net cellular glucose output [18]. However, under our culture conditions of basal insulin and supplemented leucine, phosphorylation of AKT was markedly decreased, suggesting that leucine is antagonistic to insulin, at least with respect to the regulation of AKT activity. When activated, AKT also suppresses the phosphorylation and activity of the serine/threonine kinase AMPK [21], an intracellular energy regulator influencing both glucose and lipid metabolism. Thus, with leucine supplementation the suppression of AKT would have been predicted to promote an AMPK-dependent increase in the terminal pathway effector mTORC1 [19]. However, we observed both a decrease in mTORC1 phosphorylation (leading to activation) and unchanged levels of mTORC1-dependent S6K1 phosphorylation. Collectively, then, our data suggest this seminal pathway is central to leucine-dependent glucose sensing.

Recently, AMPK regulation of the TGFβ family member *MSTN* has been linked to acute increases in glucose transport and IR [25, 26]. *MSTN* mRNA levels are elevated in peripheral tissues from obese, leptin-deficient ob/ob mice and high fat-fed wild-type mice [23] and promote glucose uptake *in vitro* by C2C12 myotubes [27]. Meanwhile, *MSTN* knock out enhances systemic insulin sensitivity and prevents obesity [28, 29]. Clinically, plasma levels of *MSTN* protein are elevated in obese patients [22, 30] and a comparison of muscle cells isolated from obese and non-obese women revealed increased *MSTN* secretion [22]. Conversely, *MSTN* mRNA levels are reduced in peripheral tissues following weight loss in mice and human patients [31, 32]. Our observations are parsimonious with these collective observations, providing perhaps the first empirical data supporting increased hepatic glucose uptake in the presence of excess extracellular leucine and suggesting that it occurs through *MSTN*-dependent AMPK modulation of glucose transporter expression.

While AMPK activates glucose transport and glycolysis in skeletal muscles, it also suppresses glycogenolysis [33], and we reported a mild reduction of glycogen in hepatic cells, but also an accumulation of fats. Under normal conditions, the excess glucose is converted into lipids carried as very-low-density lipoprotein (VLDL) and ultimately stored as TG. However, under chronic conditions of glucose uptake, pathologic levels of TG accumulation can occur and a fatty liver phenotype may develop. As described above we show leucine-dependent accumulation of *FOXO1*, which promotes transcription of microsomal TG transfer protein (MTP) and VLDL production and leading to hepatic hypertriglyceridemia [17]. With leucine supplementation we see elevated transcription of several genes involved in fatty acid synthesis, including *GPAT1*, *ACC1* and *PPARγ. GPAT1* is a key enzyme in the regulation of hepatic triglyceride biosynthesis, such that an acute reduction of mitochondrial *GPAT1* in the liver of ob/ob mice reduces triglyceride synthesis and obesity [14]. Expressed at high levels in lipogenic tissues, *ACC1* controls the regulation of long-chain fatty acids biosynthesis, and its inhibition has been proposed as a potential strategy for the treatment of obesity and related disorders [15]. In addition, *PPARγ* binds to the promoters of *SLC2A2* and *GK* [12] activating transcription, and when overexpressed leads to lipid accumulation in hepatocytes [34]. We observe analogous *in vitro* transcriptional changes, consistent with the stimulation of hepatic lipogenesis after leucine supplementation and again, these effects appear to require *MSTN*-dependent AMPK signalling. *GPAT1* expression in particular, was significantly altered by *MSTN* suppression in our experiments.

In *MSTN*-null mice, the insulin sensitivity of skeletal muscle is improved and body fat reduced [35, 36]. Further, a constitutive *MSTN* loss-of-function mutation also attenuates fat accumulation in muscle tissue and hepatic steatosis in mice fed a high-fat diet [37]. The most common cause of abnormal liver function is NAFLD [38], in which increased TG synthesis contributes to hepatic steatosis and is frequently a sequela observed with advancement of metabolic syndrome. NAFLD may even cause pancreatic β-cells to attempt compensation by increasing insulin production, leading to hyperinsulinemia and in turn, further stimulating hepatic *de novo* lipogenesis (reviewed by [39]). Emerging data from both human and animal studies support a causal role of intracellular hepatic TG accumulation in the pathogenesis of hepatic IR and human NAFLD subjects often exhibit peripheral IR as well [40].

We have also investigated a role for miRNA-mediated epigenetic effects in the manifestation of the hepatic responses to leucine exposure. MicroRNAs have now been linked to a variety of biological phenomena, and specifically to insulin secretion [41, 42], reduced viability and numbers of pancreatic β-cells [43], glucose metabolism [44] and pathological development of obesity [45]. In particular, expression of miR-143, miR-17-92b and miR-335 are significantly altered in diet-induced obese mice [46], during 3 T3-L1 adipocyte differentiation [47], and in human adipose tissues inflammation [48]. We found that suppression of miR-143 led to a strong increase in the hepatic expression of *PPARγ* and blocked the ability of leucine to induce both *MSTN* and *FOXA2* expression. Meanwhile, it has been reported that the miR-17-92 cluster, which yields six mature miRNAs including miR-92, is upregulated and promotes adipogenesis by inhibiting the key cell cycle regulator and tumor suppressor gene Rb2/p130 [47]. We found suppression of miR-92b* reduced the leucine-dependent upregulation of *MSTN*, *FOXO1*, *SLC2A2* and *FOXA2*. Further, miR-335 upregulation combines with increased expression of interleukin-6 and tumor necrosis factor-α during inflammation of human visceral adipose tissue in obesity-related IR [48], and occurs in parallel with that of *PPARγ* after the induction of 3 T3-L1 adipocyte differentiation [41]. Notably, we found that miR-335 suppression inhibited leucine-dependent increases in *FOXO1* and *SLC2A2* gene expression.

## Conclusions

In summary, we suggest that leucine may be both ‘friend’ , stimulating hepatic cell uptake of extracellular glucose, and ‘foe’ , with progression toward NAFLD-like phenotypes being perhaps the unavoidable and obligatory consequence of the enhanced glucose sequestration promoted by extracellular leucine when in pathophysiological excess. We also extend the potential functional importance of the metabolic axis of AMPK-*MSTN* signaling and miRNA mediated epigenetic mechanisms in the context of metabolic syndrome and NAFLD in particular. We also will look to extend these encouraging findings to primary cultures of hepatocytes in future studies. While out findings may offer an intriguing resolution to the apparent paradox associated with the reported pathophysiological consequences of BCAA exposures, further investigation in cultures of primary hepatocytes from clinical patients is warranted to substantiate them.

## Methods

### Chemicals and antibodies

DMEM and FBS were from Invitrogen (CA, USA). The 2-DOG was from PerkinElmer (Boston, USA). All other chemicals were from Sigma (St. Louis, MO, USA) unless otherwise stated. Antibodies for mTORC1, p(Ser^2448^)-mTORC1, p(Thr^389^)-p70-S6K1, AMPKα, p(Thr^172^)-AMPKα, AKT and p(Ser^473^)-AKT were from Cell Signaling Technology (Boston, MA, USA) and B-actin antibody was from Santa Cruz Biotechnology (Santa Cruz, CA, USA). The predesigned miRNA primers and siRNAs were from Qiagen (Hilden, Germany).

### Cell culture and treatment

The HepG2 cell line was purchased from American Type Culture Collection (Manassas, VA, USA) and were passaged in low glucose DMEM supplemented with 10% FBS at 37°C with 5% CO_2_. Cells were seeded at 10^4^ to 10^5^ cells/cm^2^ and after 24 h treated (or not) with 0.1 mM or 2.5 mM leucine for 48 h before being harvested for various assays. The survival of the cells was measured by an MTT assay after 48 h of exposure to leucine.

### Glucose uptake assay

HepG2 cells treated (or not) with leucine were washed with pre-warmed PBS twice and incubated in a glucose-free Krebs-Ringer phosphate buffer (KRP) buffer containing 1% BSA for 1.5 h at 37°C. Glucose uptake assay was then performed as described previously [49]. Results were normalized against the total intracellular protein content, which was determined by BCA assay (Thermoscientific, IL, USA).

### Glucose output assay

Glucose secreted into the medium was measured using Amplex Red Glucose Kit (Invitrogen, Carlsbad, NM, USA) according to manufacturer’s instructions. Following leucine treatment (or not), cells were washed twice with prewarmed PBS and incubated for 1.5 h in glucose production assay medium (glucose and phenol red-free DMEM containing 2 mM sodium pyruvate and 20 mM sodium lactate). Next, 1 nM insulin was added 10 min before the end of the incubation period as appropriate. Media was collected for analysis. Data were normalized against total intracellular protein.

### Triglyceride measurement

Total intracellular TG content was measured using a fluorometric method kit (BioVision, CA, USA) in accordance with the manufacturer’s instructions. Data were normalized against total intracellular protein.

### Glycogen measurement

The assay was performed using a Glycogen Assay Kit (BioVision, CA, USA) according to the manufacturer’s instructions. Glycogen content was normalized against the total intracellular protein.

### Real-time PCR

Total RNA was isolated from leucine-treated (or not) HepG2 cells using PureLink RNA Mini Kit (Invitrogen, CA, USA) and cDNA was synthesized from 2 μg of total RNA using Transcriptor First Strand Synthesis kit (Roche, Mannheim, Germany). qPCR analysis was carried out on a LightCycler-480 II (Roche, Switzerland) in 10 μl volumes containing Light Cycler 480 SYBR Green, 0.5 mM of reverse or forward PCR primers (Table 2) and 1 μl of first-strand cDNA. The endogenous control peptidyl-prolyl isomerase A (*PPIA*) gene expression was chosen as the housekeeping gene as its threshold was constant across different conditions. The mRNA expression levels were normalized against *PPIA* by subtracting its average cycle threshold from the average threshold for each cDNA sample yielding a level of mRNA expression for the target molecule relative to the endogenous RNA reference gene.

**Table 2:** **Primer sequences used in real-time PCR (qPCR)**

Gene name	Primers	Primer sequences
***SLC2A2***	**Sense**	5'- CATTCCAATTAGAAAGAGAGAACGTC-3'
	**Antisense**	5'-AGCAAACCTGTTTATGCAACC-3'
***G6Pase***	**Sense**	5'-TACGTCCTCTTCCCCATCTG-3'
	**Antisense**	5'-CCTGGTCCAGTCTCACAGGT-3'
***PCK1***	**Sense**	5'-GGTTCCCAGGGTGCATGAAA-3'
	**Antisense**	5'-CACGTAGGGTGAATCCGTCAG-3'-3'
***PC***	**Sense**	5'-TTGCCCACTTCAAGGACTTC-3'
	**Antisense**	5'-CTTTGATGTGCAGCGTCTTG-3'
***FOXO1***	**Sense**	5’-GCTGCATCCATGGACAACAACA-3'
	**Antisense**	5’-CGAGGGCGAAATGTACTCCAGTT-3'
***PPARGC1A***	**Sense**	5'-TGTGCAACTCTCTGGAACTG-3'
	**Antisense**	5'-TGAGGACTTGCTGAGTGGTG-3'
***MSTN***	**Sense**	5'-CGTCTGGAAACAGCTCCTAACA-3'
	**Antisense**	5'-GAAAATCAGACTCTGTAGGCATGGT-3'
***GPAT1***	**Sense**	5’-AACCCCAGTATCCCGTCTTT-3’
	**Antisense**	5’-CAGTCACATTGGTGGCAAAC-3’
***FOXA2***	**Sense**	5-TGTTCATGCCGTTCATCCC-3
	**Antisense**	5-GGAGCGGTGAAGATGGAAG-3
***GK***	**Sense**	5'-GATGCACTCAGAGATGTAGTCG-3'
	**Antisense**	5'-TGAAGGTGGGAGAAGGTGAG-3'
***ACC1***	**Sense**	5’-ATCCCG TACCTTCTTCTACTG-3’
	**Antisense**	5’-CCCAAACATAAGCCTTCACTG-3’
***PPAR*** *γ*	**Sense**	5'-CCACTATGGAGTTCATGCTTGTGAAGG-3'
	**Antisense**	5'-TGCAGCGGGGTGATGTGTTTGAACTTG-3'
***PPIA***	**Sense**	5'-TCTTGAGGGAAGCATATTGG-3'
	**Antisense**	5'-CAGGGAGACTGACTGTAGCAC-3'

### Western blotting

HepG2 cells (treated or not) were washed twice with ice-cold PBS. Ice-cold TK lysis buffer was added containing protease and phosphatase inhibitors. A total of 20 μg protein from whole cell lysates was resolved using 10% SDS-PAGE followed by transfer onto Immobilon-P PVDF membrane (Millipore, MA, USA). Primary and secondary antibodies were diluted in 2% skim-milk/PBS-0.1% Tween 20.

### Reverse transfection

*MSTN* siRNA (4392420-s5679, Invitrogen, CA, USA) or control oligonucleotides (4390843, Invitrogen, CA, USA) were reverse transfected into HepG2 cells in a 24-well plate using Lipofectamine RNAiMAX (Invitrogen, CA, USA). Briefly, Lipofectamine and diluted siRNA were added to Opti-MEM I Medium (Invitrogen, NY, USA) and incubated in wells for 20 min. Cells were then added at a density of 10^5^ cells/well and 24 h later treated with leucine for a further 48 h before being harvested.

### miRNA microarray

A PureLink RNA Mini Kit was used to extract total RNA (Invitrogen, CA, USA). The RNA was initially evaluated by 260/280 ratio using a NanoDrop ND-1000 Spectrophotometer (NanoDrop Technologies, DE, USA) and was further assessed on Agilent 2100 Bioanalyzer (Agilent Technologies, CA, USA) after preparation with an Agilent RNA 6000 Nano kit. All samples showed values of 260/280 above 1.8 and RIN scores of at least 8.0. Reverse transcription was carried out using 500 ng total RNA. Then, cRNA was labeled with Biotin using Affymetrix Flash Tag Biotin HSR RNA Labeling kit. The fragmented-Biotin-labeled cRNA was then added to the array (Affymetrix GeneChip miRNA 3.0), and after washing to remove any unbound RNA , hybridization was assessed by fluorescent staining (GeneChip Hybridization, Wash and Stain kit) and scanning with a GeneChip Scanner 3000 system. Robust multi-array average (RMA) background correction and quartile normalization were used to adjust signal intensity data. All data then were converted into log2 values for further statistical analysis.

### Validation of miRNA expression/ gene targets

Candidate targets for validation by qPCR were determined according to both fold-change and significance at *P* ≤0.05. Also, miRBase (http://microrna.sanger.ac.uk/) and miRWalk (http://www.umm.uni-heidelberg.de/apps/zmf/mirwalk/index.html) databases were used to identify potential and validated gene targets. The cDNA synthesis was carried out with 2 μg total RNA using a miScript II RT Kit (Qiagen, Hilden, Germany) , and expression of the miR-143, miR-92b*, miR-335, miR-181d, miR-3185 and miR-4763 was assayed with a a miScript SYBR Green PCR kit (Qiagen, Hilden, Germany). Data was normalized to RNU6-2 snRNA expression. Commercially available siRNAs were used to inhibit differentially expressed miRNAs (Qiagen, Hilden, Germany) following qPCR to assess target gene effects.

### Statistical analysis

All analyses were performed using the IBM SPSS statistical program (version 21, NY, USA). All results are presented as mean ± SEM from at least three independent experiments done in triplicates. A one-way ANOVA with a Tukey post-hoc test was used to assess differences between groups. *P* ≤0.05 was considered statistically significant.

## Abbreviations

*ACC1*: acetyl-CoA carboxylase
AKT: protein kinase B
AMPK: AMP-activated protein kinase
BCAAs: branched-chain amino acids
*FOXO1*: forkhead transcription factor
*FOXA2*: forkhead box protein A2
*GK*: glucokinase
*GPAT1*: glycerol-3-phosphate acyltransferase
*G6Pase*: glucose 6-phosphatase
IR: insulin resistance
KRP: Krebs-Ringer phosphate buffer
*MSTN*: myostatin
mTORC1: mammalian target of rapamycine complex 1
NAFLD: nonalcoholic fatty liver disease
*PCK1*: phosphoenolpyruvate carboxykinase (PEPCK)
*PC*: pyruvate carboxylase
*PPARGC1A*: peroxisome proliferative activated receptor-γ co-activator 1
*PPARγ*: peroxisome proliferator-activated receptor gamma
*PPIA*: peptidyl-prolyl isomerase A
qPCR: real-time PCR
S6K1: ribosomal protein S6 kinase beta-1
RMA: robust multi-array average
*SLC2A2*: solute carrier family member2
TG: triglyceride
T2DM: type 2 diabetes mellitus
VLDL: very-low-density lipoprotein
2-DOG: 2-deoxy-D-[1,2-^3^H] glucose.
